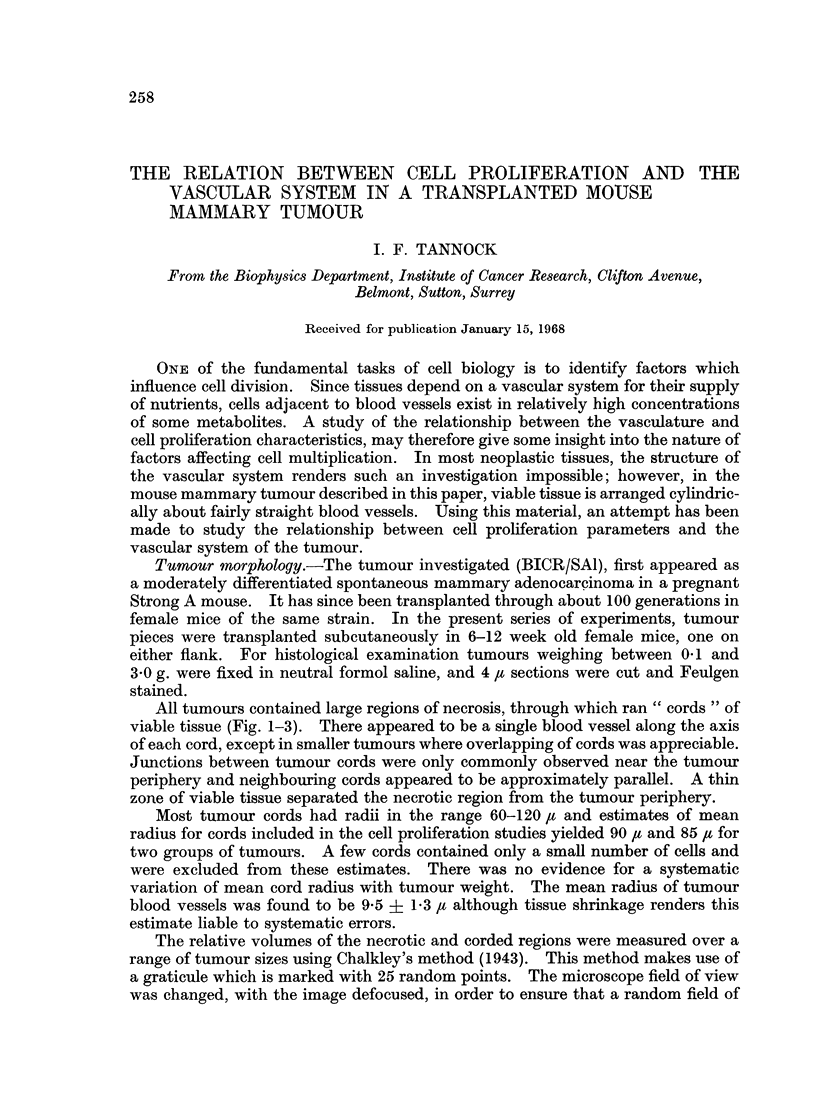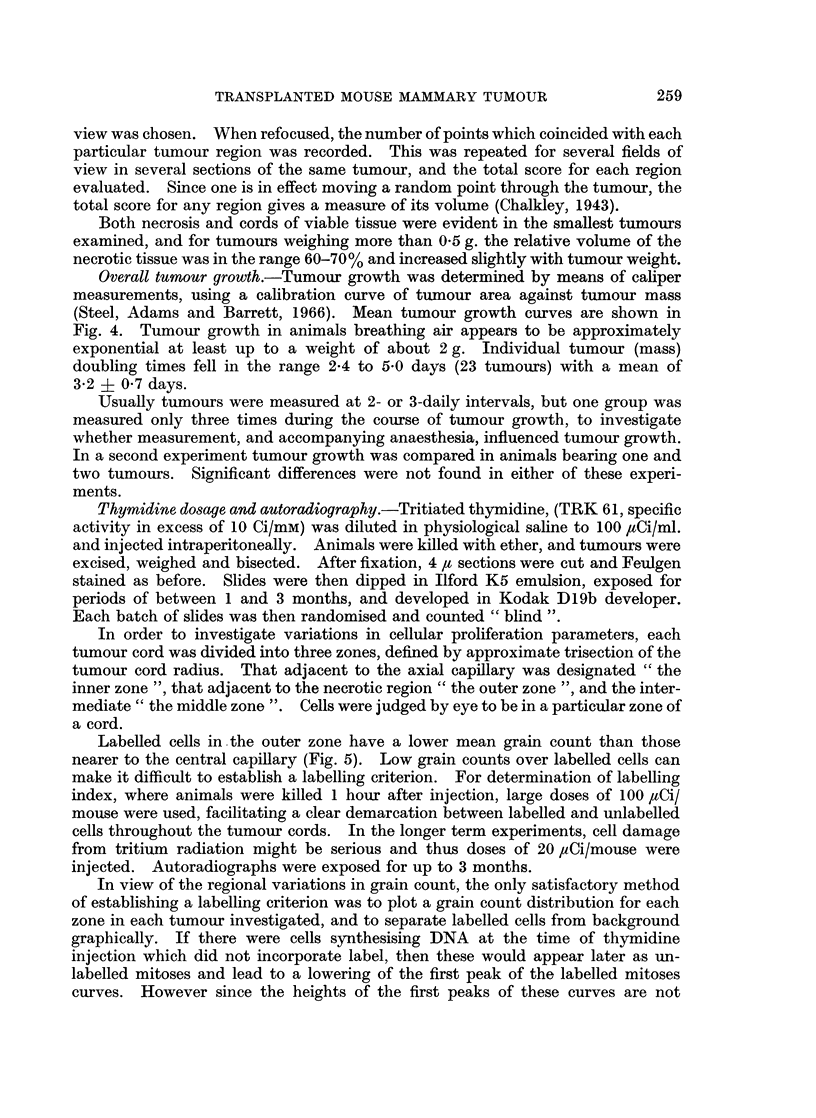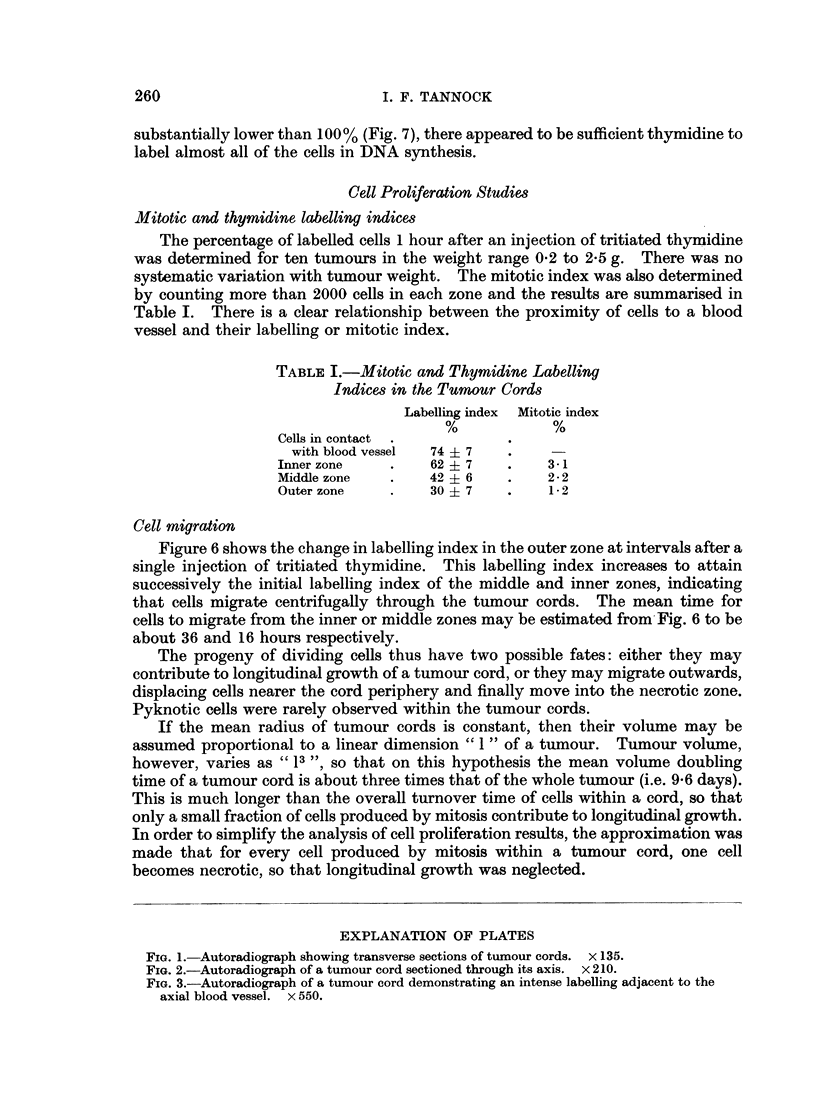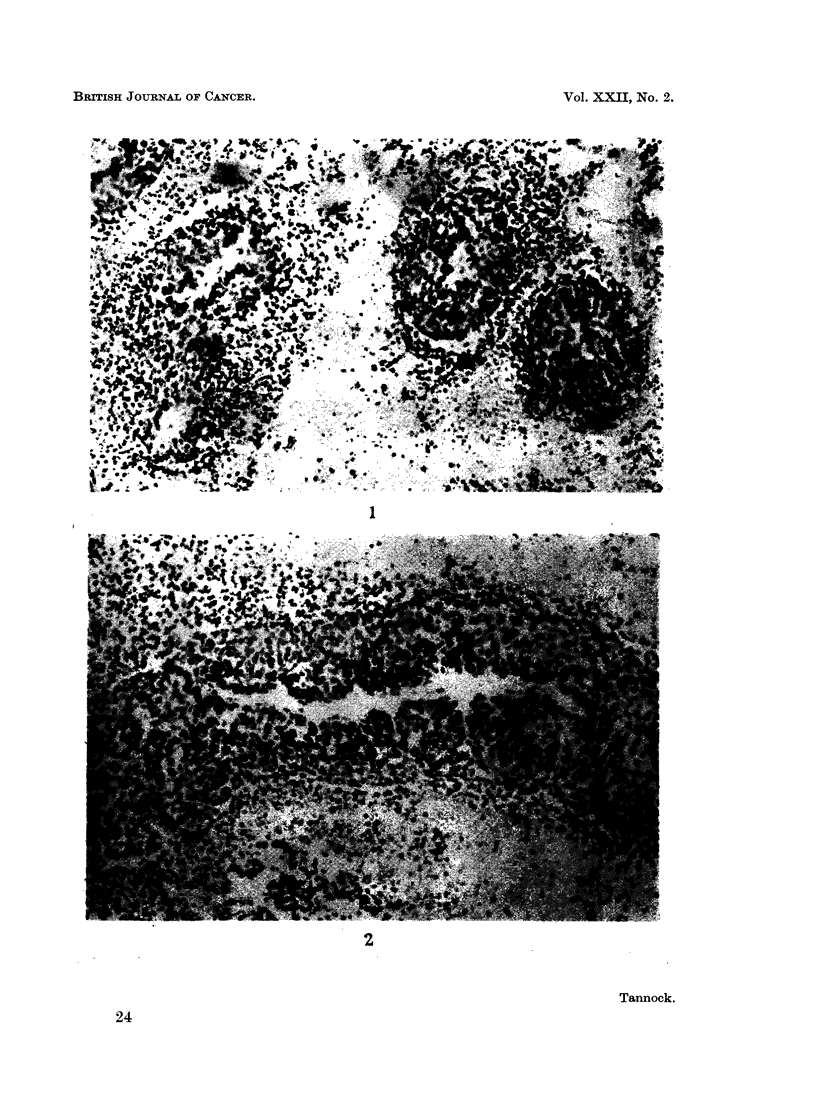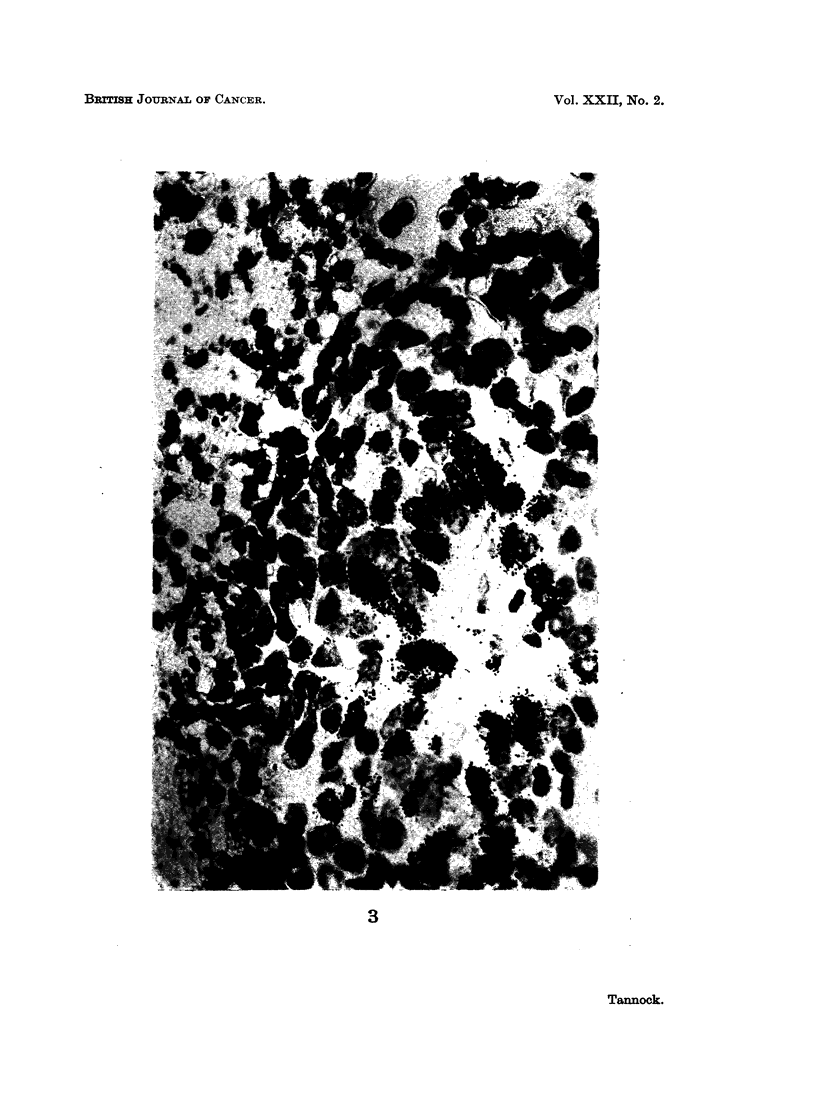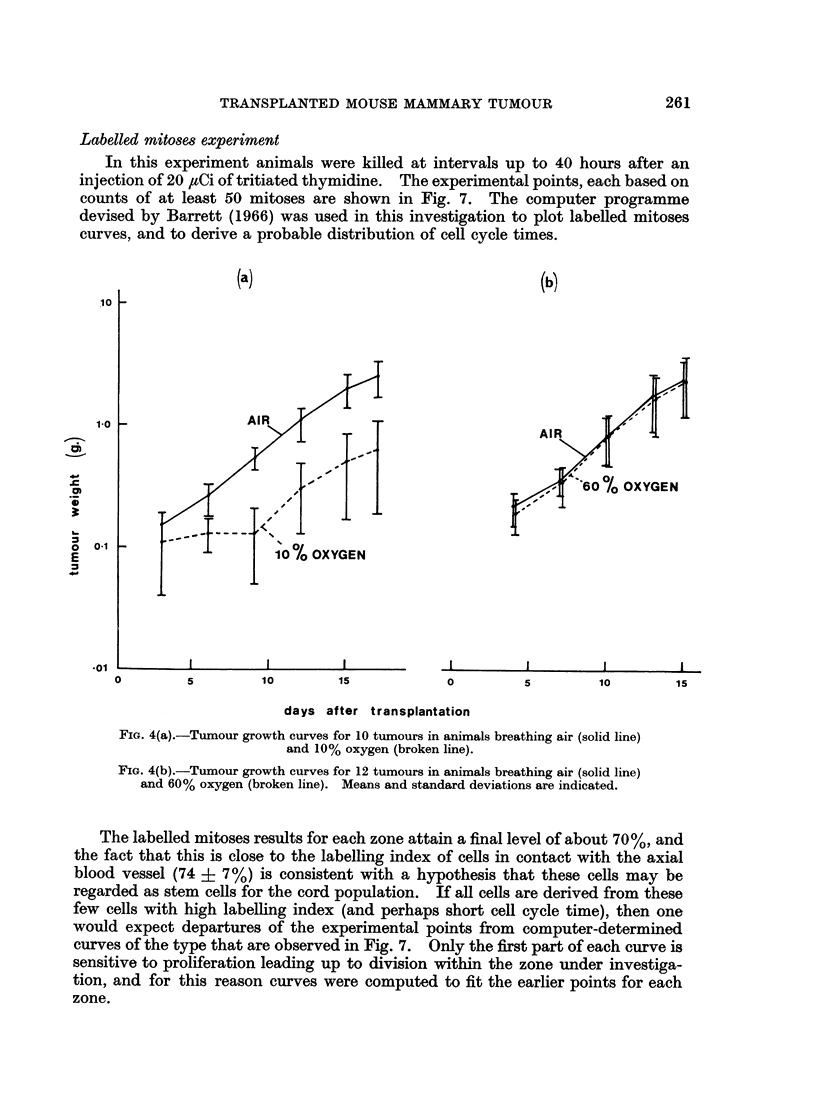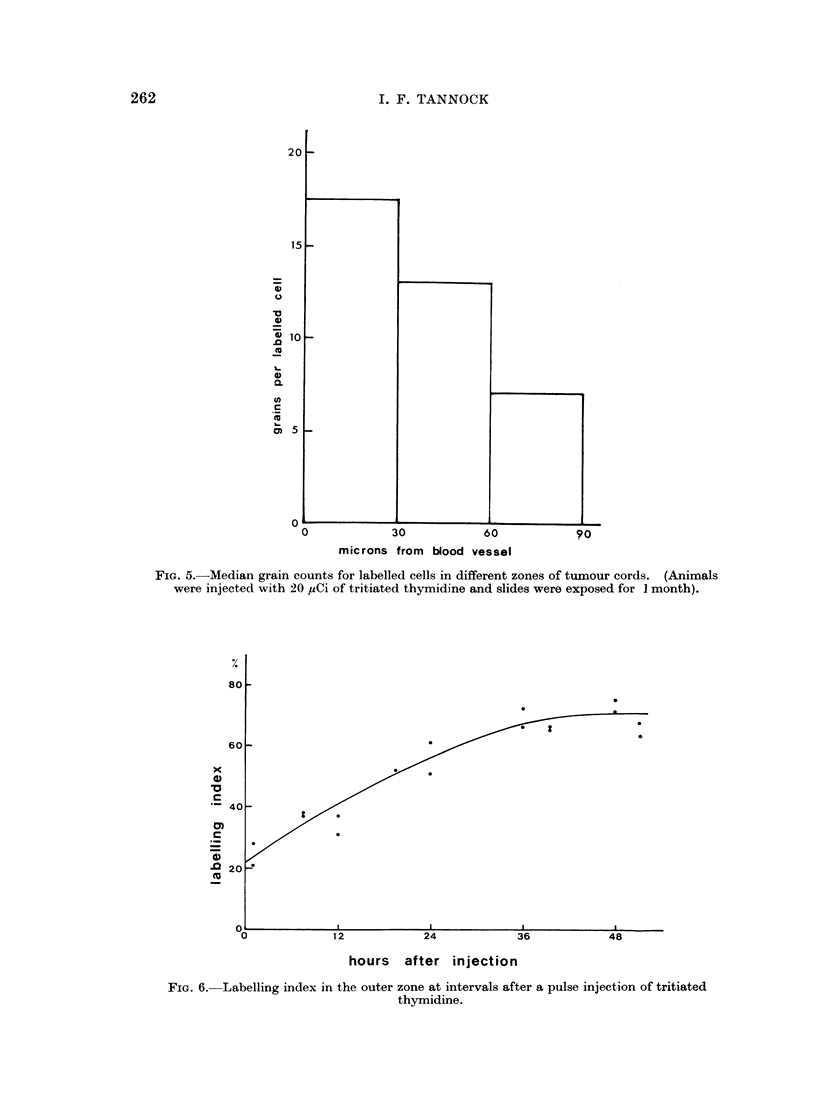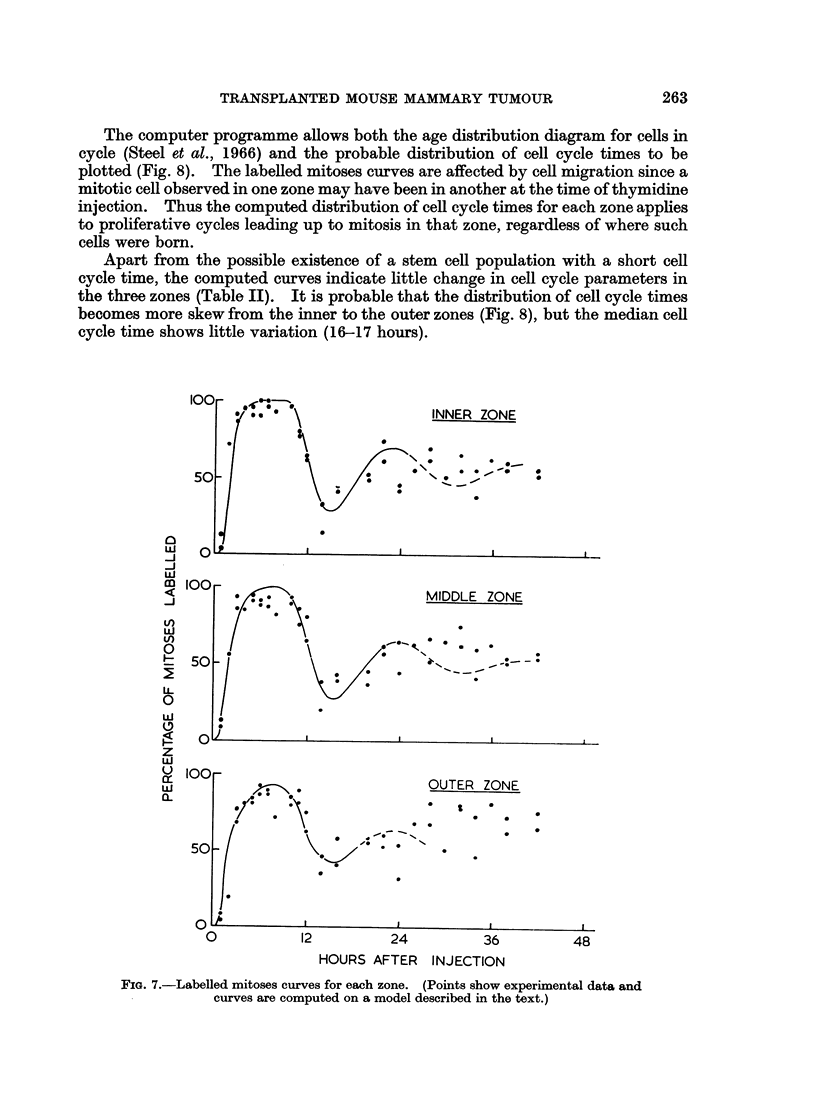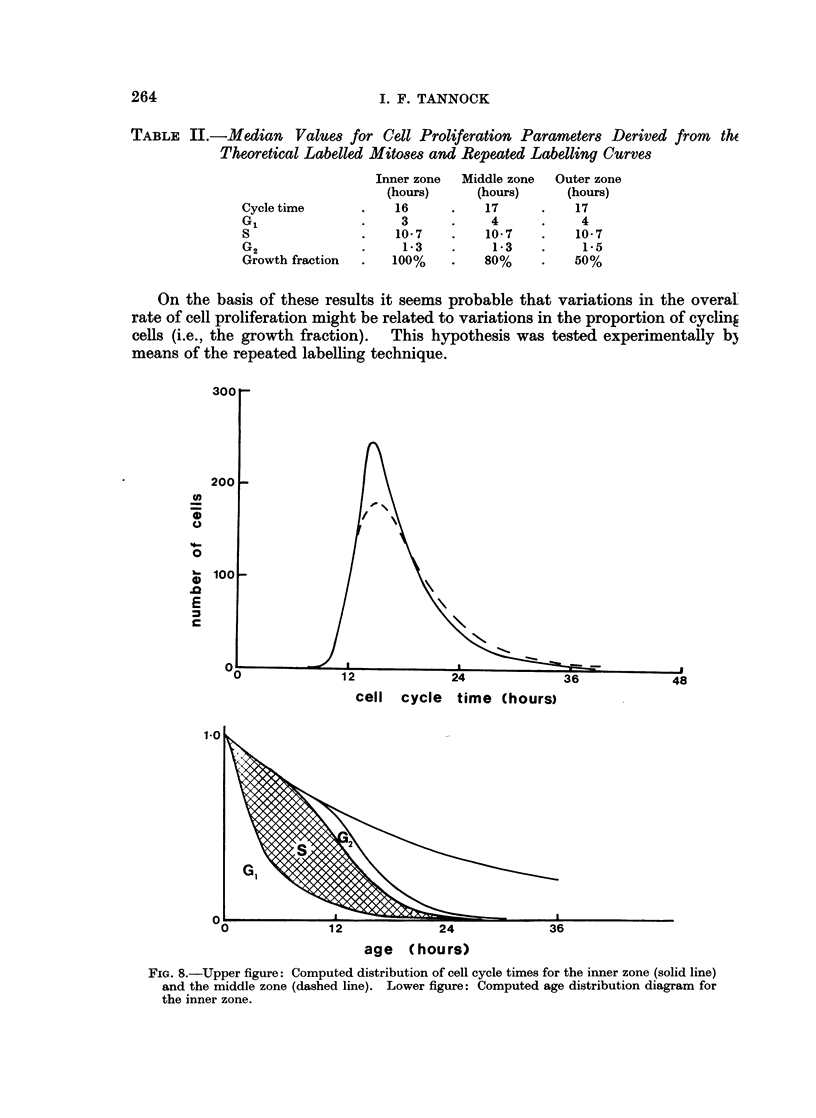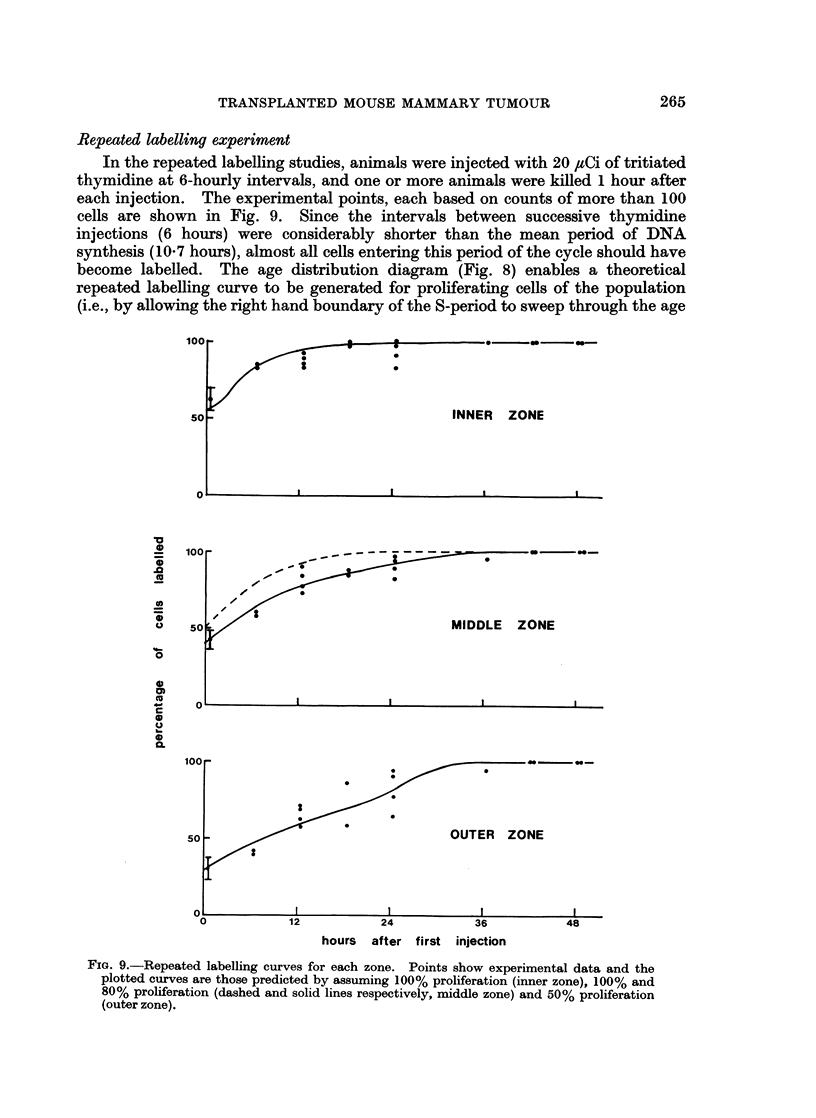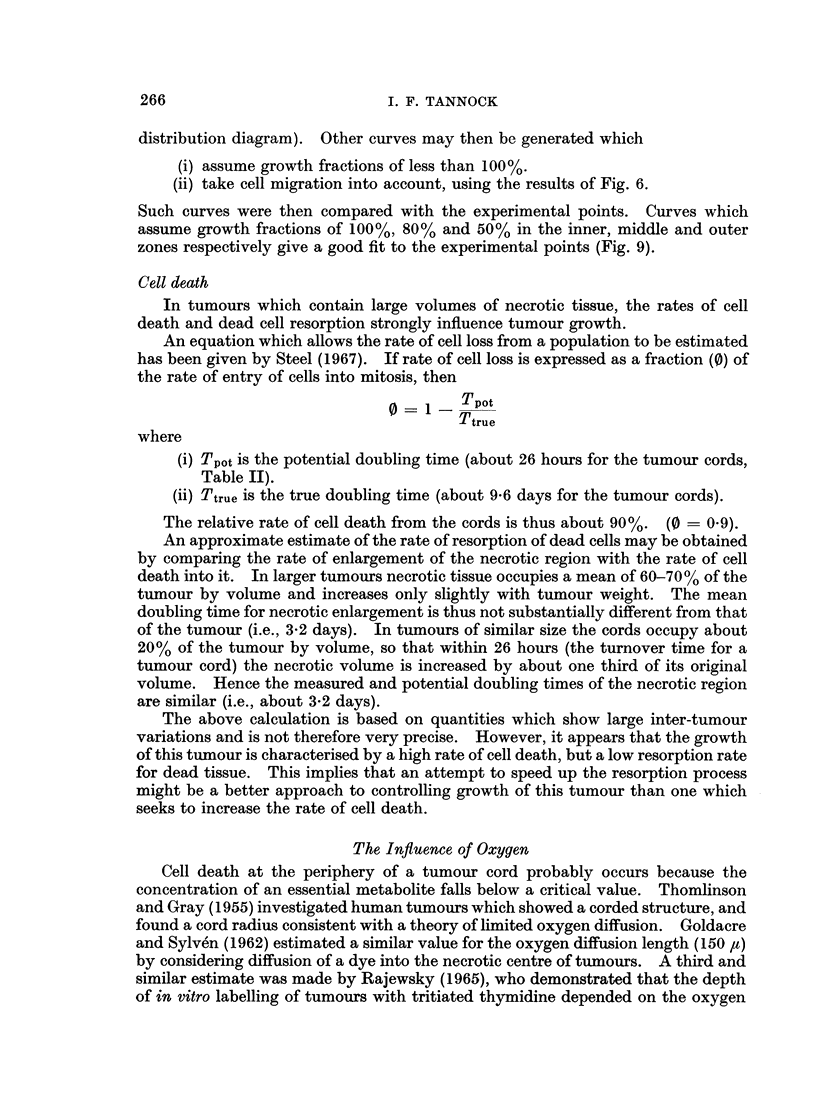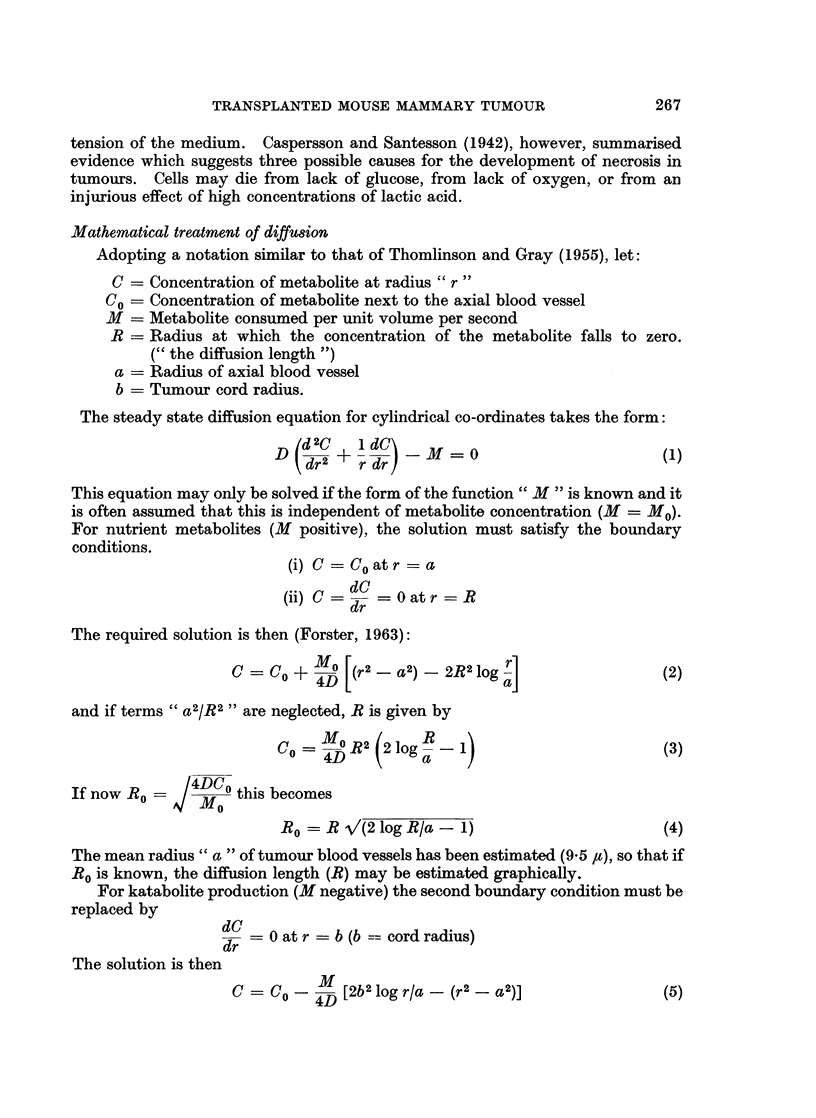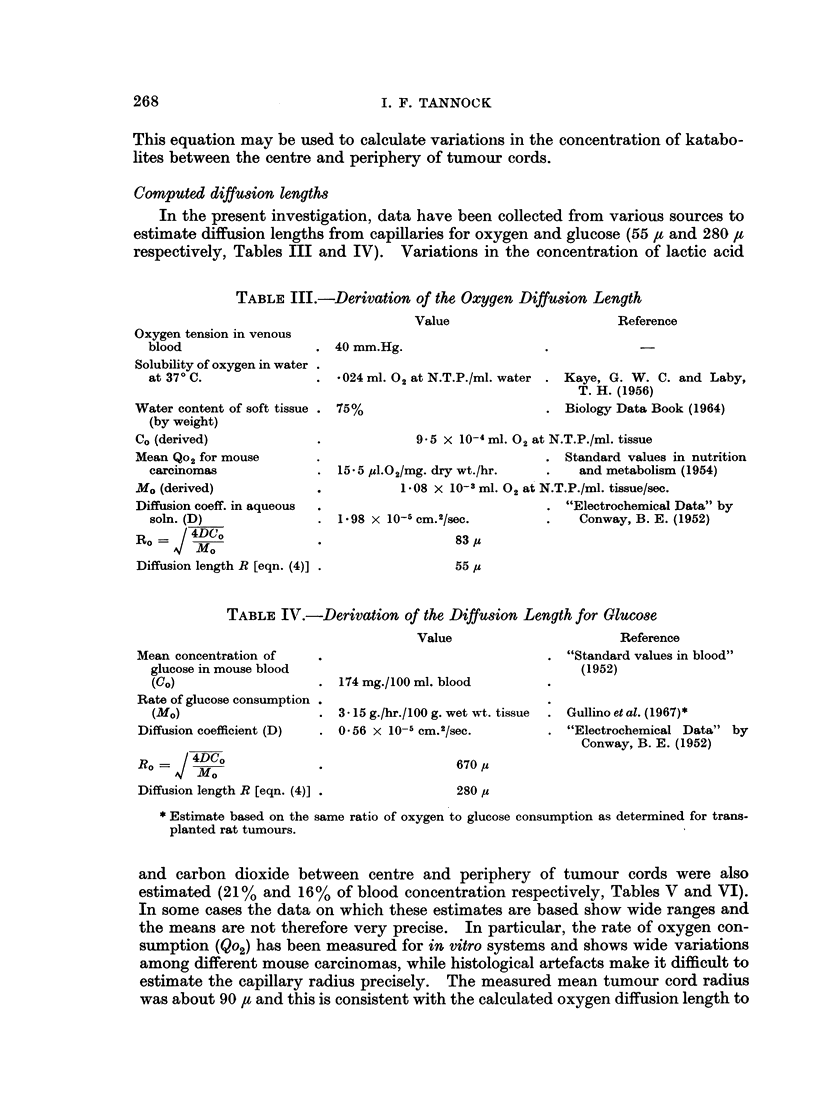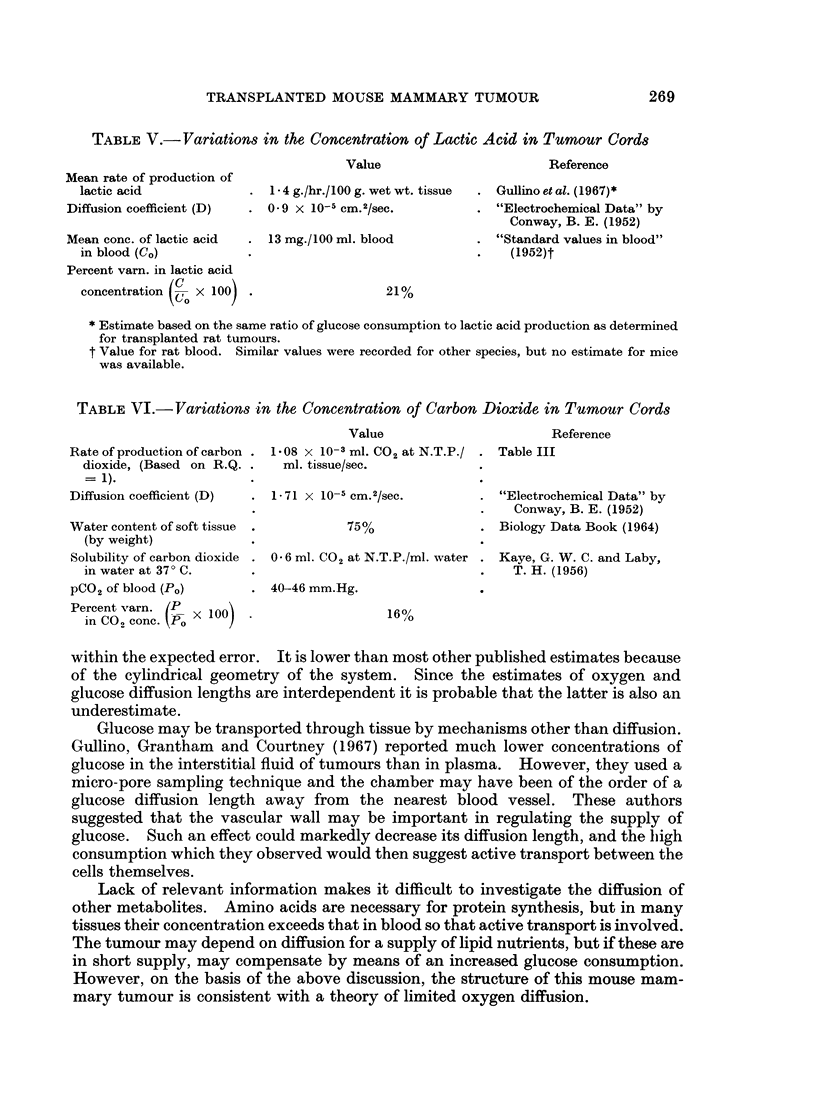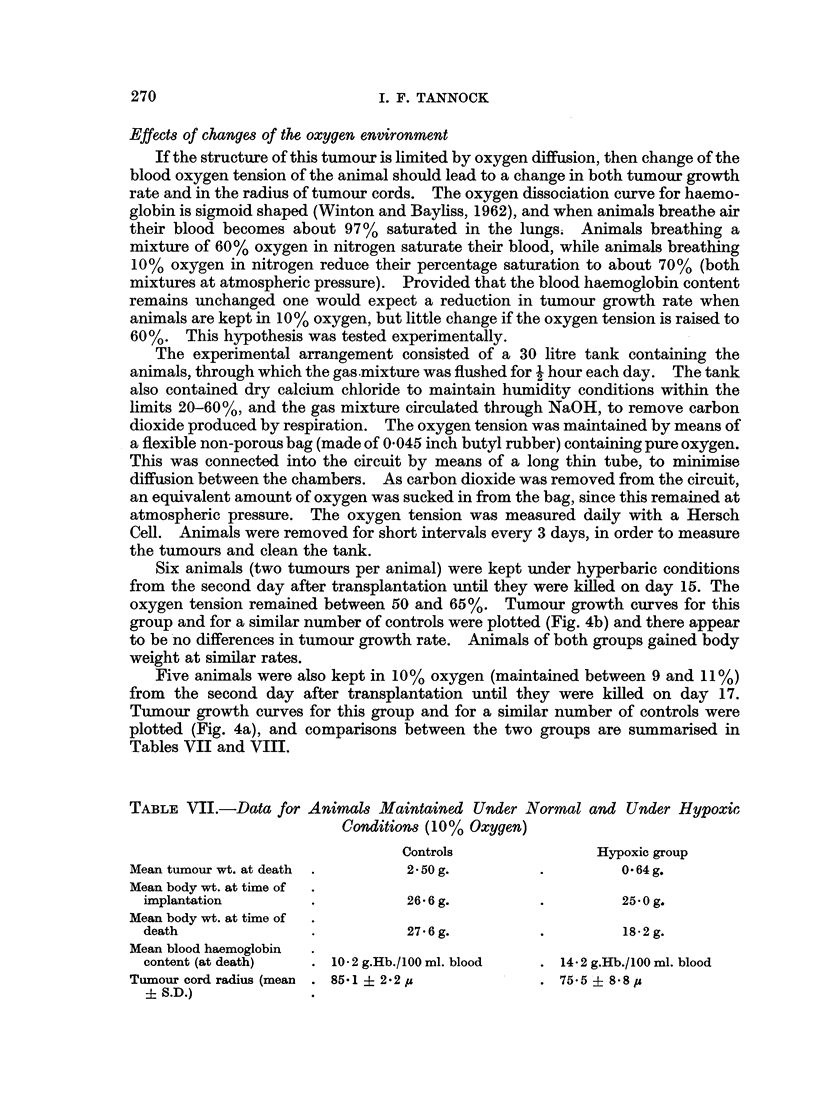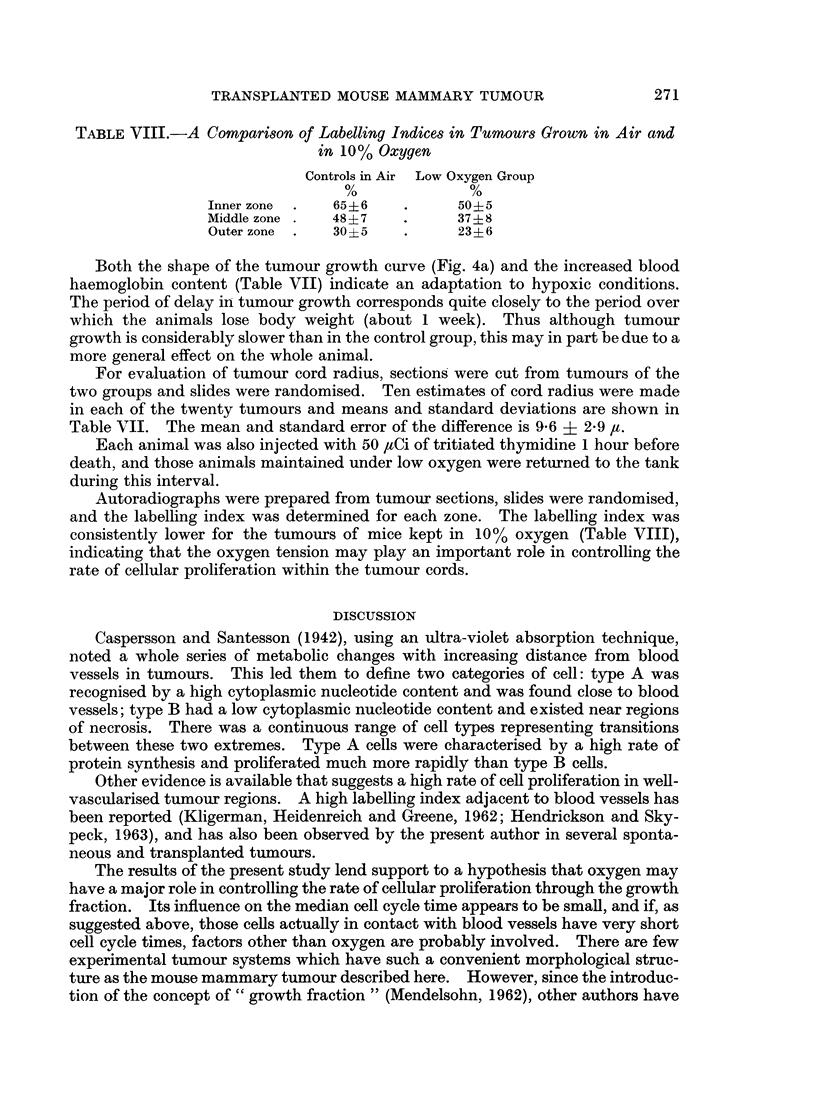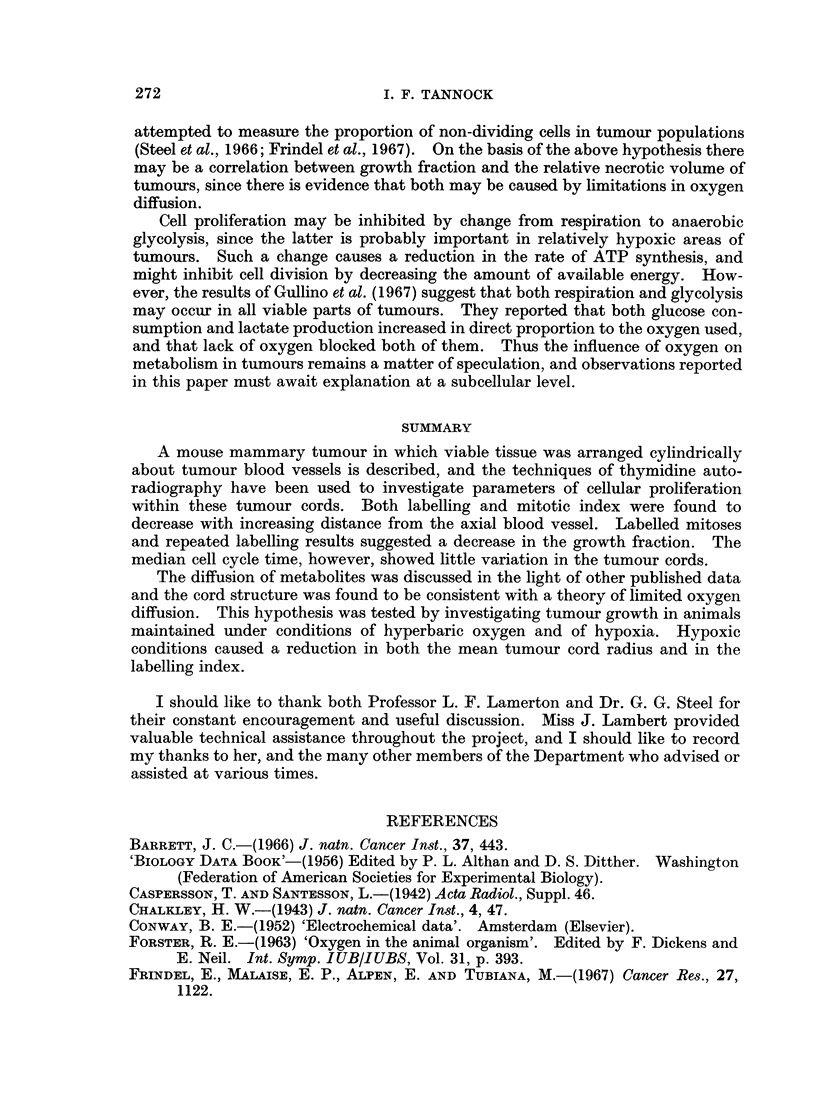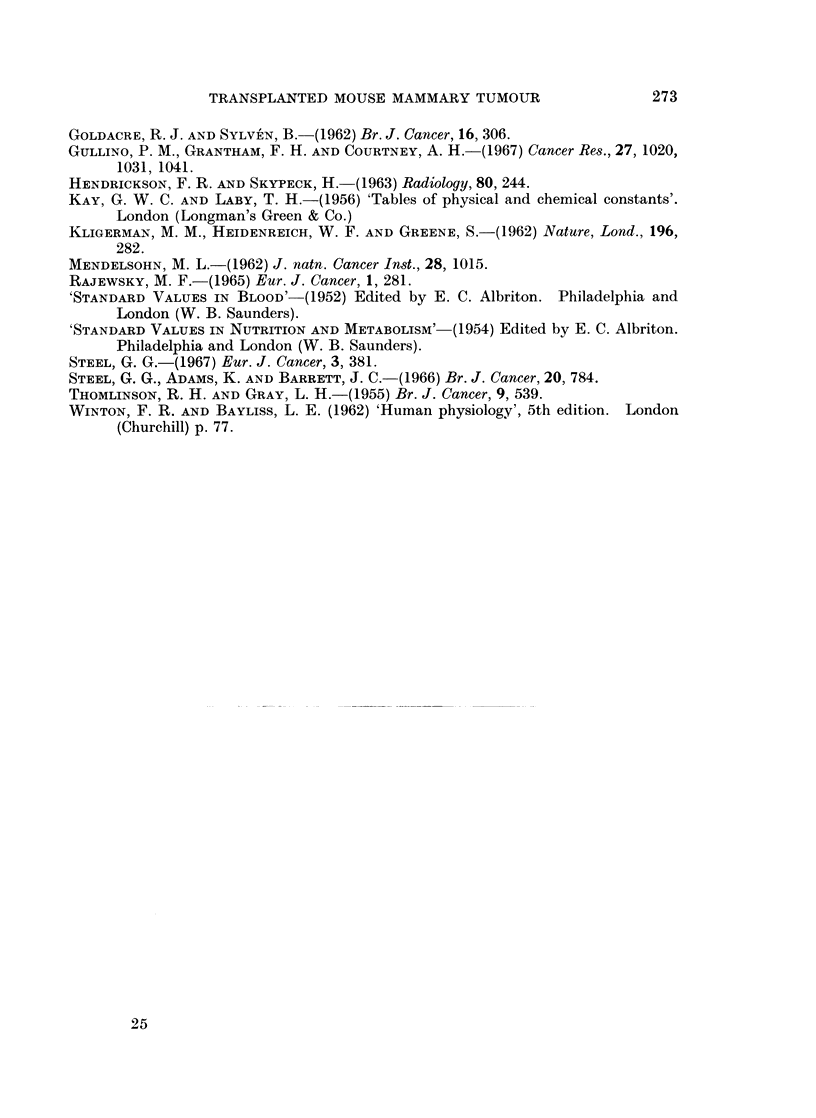# The relation between cell proliferation and the vascular system in a transplanted mouse mammary tumour.

**DOI:** 10.1038/bjc.1968.34

**Published:** 1968-06

**Authors:** I. F. Tannock

## Abstract

**Images:**


### THE RELATION BETWEEN CELL PROLIFERATION AND THE VASCULAR SYSTEM IN A TRANSPLANTED MOUSE MAMMARY TUMOUR

I. F. TANNOCK

*From the Biophysics Department, Institute of Cancer Research, Clifton Avenue, Belmont, Sutton, Surrey*

Received for publication January 15, 1968

ONE of the fundamental tasks of cell biology is to identify factors which influence cell division. Since tissues depend on a vascular system for their supply of nutrients, cells adjacent to blood vessels exist in relatively high concentrations of some metabolites. A study of the relationship between the vasculature and cell proliferation characteristics, may therefore give some insight into the nature of factors affecting cell multiplication. In most neoplastic tissues, the structure of the vascular system renders such an investigation impossible; however, in the mouse mammary tumour described in this paper, viable tissue is arranged cylindrically about fairly straight blood vessels. Using this material, an attempt has been made to study the relationship between cell proliferation parameters and the vascular system of the tumour.

*Tumour morphology.*—The tumour investigated (BICR/SA1), first appeared as a moderately differentiated spontaneous mammary adenocarcinoma in a pregnant Strong A mouse. It has since been transplanted through about 100 generations in female mice of the same strain. In the present series of experiments, tumour pieces were transplanted subcutaneously in 6–12 week old female mice, one on either flank. For histological examination tumours weighing between 0·1 and 3·0 g. were fixed in neutral formol saline, and 4 $\mu$ sections were cut and Feulgen stained.

All tumours contained large regions of necrosis, through which ran " cords " of viable tissue (Fig. 1–3). There appeared to be a single blood vessel along the axis of each cord, except in smaller tumours where overlapping of cords was appreciable. Junctions between tumour cords were only commonly observed near the tumour periphery and neighbouring cords appeared to be approximately parallel. A thin zone of viable tissue separated the necrotic region from the tumour periphery.

Most tumour cords had radii in the range 60–120 $\mu$ and estimates of mean radius for cords included in the cell proliferation studies yielded 90 $\mu$ and 85 $\mu$ for two groups of tumours. A few cords contained only a small number of cells and were excluded from these estimates. There was no evidence for a systematic variation of mean cord radius with tumour weight. The mean radius of tumour blood vessels was found to be $9\cdot5 \pm 1\cdot3$ $\mu$ although tissue shrinkage renders this estimate liable to systematic errors.

The relative volumes of the necrotic and corded regions were measured over a range of tumour sizes using Chalkley's method (1943). This method makes use of a graticule which is marked with 25 random points. The microscope field of view was changed, with the image defocused, in order to ensure that a random field of view was chosen. When refocused, the number of points which coincided with each particular tumour region was recorded. This was repeated for several fields of view in several sections of the same tumour, and the total score for each region evaluated. Since one is in effect moving a random point through the tumour, the total score for any region gives a measure of its volume (Chalkley, 1943).

Both necrosis and cords of viable tissue were evident in the smallest tumours examined, and for tumours weighing more than 0·5 g. the relative volume of the necrotic tissue was in the range 60–70% and increased slightly with tumour weight.

*Overall tumour growth.*—Tumour growth was determined by means of caliper measurements, using a calibration curve of tumour area against tumour mass (Steel, Adams and Barrett, 1966). Mean tumour growth curves are shown in Fig. 4. Tumour growth in animals breathing air appears to be approximately exponential at least up to a weight of about 2 g. Individual tumour (mass) doubling times fell in the range 2·4 to 5·0 days (23 tumours) with a mean of $3 \cdot 2 \pm 0 \cdot 7$ days.

Usually tumours were measured at 2- or 3-daily intervals, but one group was measured only three times during the course of tumour growth, to investigate whether measurement, and accompanying anaesthesia, influenced tumour growth. In a second experiment tumour growth was compared in animals bearing one and two tumours. Significant differences were not found in either of these experiments.

*Thymidine dosage and autoradiography.*—Tritiated thymidine, (TRK 61, specific activity in excess of 10 Ci/mM) was diluted in physiological saline to 100 μCi/ml. and injected intraperitoneally. Animals were killed with ether, and tumours were excised, weighed and bisected. After fixation, 4 μ sections were cut and Feulgen stained as before. Slides were then dipped in Ilford K5 emulsion, exposed for periods of between 1 and 3 months, and developed in Kodak D19b developer. Each batch of slides was then randomised and counted " blind ".

In order to investigate variations in cellular proliferation parameters, each tumour cord was divided into three zones, defined by approximate trisection of the tumour cord radius. That adjacent to the axial capillary was designated " the inner zone ", that adjacent to the necrotic region " the outer zone ", and the intermediate " the middle zone ". Cells were judged by eye to be in a particular zone of a cord.

Labelled cells in the outer zone have a lower mean grain count than those nearer to the central capillary (Fig. 5). Low grain counts over labelled cells can make it difficult to establish a labelling criterion. For determination of labelling index, where animals were killed 1 hour after injection, large doses of 100 μCi/mouse were used, facilitating a clear demarcation between labelled and unlabelled cells throughout the tumour cords. In the longer term experiments, cell damage from tritium radiation might be serious and thus doses of 20 μCi/mouse were injected. Autoradiographs were exposed for up to 3 months.

In view of the regional variations in grain count, the only satisfactory method of establishing a labelling criterion was to plot a grain count distribution for each zone in each tumour investigated, and to separate labelled cells from background graphically. If there were cells synthesising DNA at the time of thymidine injection which did not incorporate label, then these would appear later as unlabelled mitoses and lead to a lowering of the first peak of the labelled mitoses curves. However since the heights of the first peaks of these curves are not substantially lower than 100% (Fig. 7), there appeared to be sufficient thymidine to label almost all of the cells in DNA synthesis.

### *Cell Proliferation Studies*

### *Mitotic and thymidine labelling indices*

The percentage of labelled cells 1 hour after an injection of tritiated thymidine was determined for ten tumours in the weight range 0·2 to 2·5 g. There was no systematic variation with tumour weight. The mitotic index was also determined by counting more than 2000 cells in each zone and the results are summarised in Table I. There is a clear relationship between the proximity of cells to a blood vessel and their labelling or mitotic index.

TABLE I.—*Mitotic and Thymidine Labelling Indices in the Tumour Cords*

| | Labelling index % | Mitotic index % |
|---|---|---|
| Cells in contact with blood vessel | 74 ± 7 | — |
| Inner zone | 62 ± 7 | 3·1 |
| Middle zone | 42 ± 6 | 2·2 |
| Outer zone | 30 ± 7 | 1·2 |

### *Cell migration*

Figure 6 shows the change in labelling index in the outer zone at intervals after a single injection of tritiated thymidine. This labelling index increases to attain successively the initial labelling index of the middle and inner zones, indicating that cells migrate centrifugally through the tumour cords. The mean time for cells to migrate from the inner or middle zones may be estimated from Fig. 6 to be about 36 and 16 hours respectively.

The progeny of dividing cells thus have two possible fates: either they may contribute to longitudinal growth of a tumour cord, or they may migrate outwards, displacing cells nearer the cord periphery and finally move into the necrotic zone. Pyknotic cells were rarely observed within the tumour cords.

If the mean radius of tumour cords is constant, then their volume may be assumed proportional to a linear dimension " l " of a tumour. Tumour volume, however, varies as " $l^3$ ", so that on this hypothesis the mean volume doubling time of a tumour cord is about three times that of the whole tumour (i.e. 9·6 days). This is much longer than the overall turnover time of cells within a cord, so that only a small fraction of cells produced by mitosis contribute to longitudinal growth. In order to simplify the analysis of cell proliferation results, the approximation was made that for every cell produced by mitosis within a tumour cord, one cell becomes necrotic, so that longitudinal growth was neglected.

---

EXPLANATION OF PLATES

FIG. 1.—Autoradiograph showing transverse sections of tumour cords. ×135.
FIG. 2.—Autoradiograph of a tumour cord sectioned through its axis. ×210.
FIG. 3.—Autoradiograph of a tumour cord demonstrating an intense labelling adjacent to the axial blood vessel. ×550.

1

2

Tannock.

3

Tannock.

*Labelled mitoses experiment*

In this experiment animals were killed at intervals up to 40 hours after an injection of 20 $\mu$Ci of tritiated thymidine. The experimental points, each based on counts of at least 50 mitoses are shown in Fig. 7. The computer programme devised by Barrett (1966) was used in this investigation to plot labelled mitoses curves, and to derive a probable distribution of cell cycle times.

FIG. 4(a).—Tumour growth curves for 10 tumours in animals breathing air (solid line) and 10% oxygen (broken line).

FIG. 4(b).—Tumour growth curves for 12 tumours in animals breathing air (solid line) and 60% oxygen (broken line). Means and standard deviations are indicated.

The labelled mitoses results for each zone attain a final level of about 70%, and the fact that this is close to the labelling index of cells in contact with the axial blood vessel (74 $\pm$ 7%) is consistent with a hypothesis that these cells may be regarded as stem cells for the cord population. If all cells are derived from these few cells with high labelling index (and perhaps short cell cycle time), then one would expect departures of the experimental points from computer-determined curves of the type that are observed in Fig. 7. Only the first part of each curve is sensitive to proliferation leading up to division within the zone under investigation, and for this reason curves were computed to fit the earlier points for each zone.

FIG. 5.—Median grain counts for labelled cells in different zones of tumour cords. (Animals were injected with 20 $\mu$Ci of tritiated thymidine and slides were exposed for 1 month).

FIG. 6.—Labelling index in the outer zone at intervals after a pulse injection of tritiated thymidine.

The computer programme allows both the age distribution diagram for cells in cycle (Steel *et al.*, 1966) and the probable distribution of cell cycle times to be plotted (Fig. 8). The labelled mitoses curves are affected by cell migration since a mitotic cell observed in one zone may have been in another at the time of thymidine injection. Thus the computed distribution of cell cycle times for each zone applies to proliferative cycles leading up to mitosis in that zone, regardless of where such cells were born.

Apart from the possible existence of a stem cell population with a short cell cycle time, the computed curves indicate little change in cell cycle parameters in the three zones (Table II). It is probable that the distribution of cell cycle times becomes more skew from the inner to the outer zones (Fig. 8), but the median cell cycle time shows little variation (16–17 hours).

FIG. 7.—Labelled mitoses curves for each zone. (Points show experimental data and curves are computed on a model described in the text.)

TABLE II.—*Median Values for Cell Proliferation Parameters Derived from the Theoretical Labelled Mitoses and Repeated Labelling Curves*

| | Inner zone (hours) | Middle zone (hours) | Outer zone (hours) |
|---|---|---|---|
| Cycle time | 16 | 17 | 17 |
| $G_1$ | 3 | 4 | 4 |
| S | 10·7 | 10·7 | 10·7 |
| $G_2$ | 1·3 | 1·3 | 1·5 |
| Growth fraction | 100% | 80% | 50% |

On the basis of these results it seems probable that variations in the overall rate of cell proliferation might be related to variations in the proportion of cycling cells (i.e., the growth fraction). This hypothesis was tested experimentally by means of the repeated labelling technique.

FIG. 8.—Upper figure: Computed distribution of cell cycle times for the inner zone (solid line) and the middle zone (dashed line). Lower figure: Computed age distribution diagram for the inner zone.

*Repeated labelling experiment*

In the repeated labelling studies, animals were injected with 20 $\mu$Ci of tritiated thymidine at 6-hourly intervals, and one or more animals were killed 1 hour after each injection. The experimental points, each based on counts of more than 100 cells are shown in Fig. 9. Since the intervals between successive thymidine injections (6 hours) were considerably shorter than the mean period of DNA synthesis (10·7 hours), almost all cells entering this period of the cycle should have become labelled. The age distribution diagram (Fig. 8) enables a theoretical repeated labelling curve to be generated for proliferating cells of the population (i.e., by allowing the right hand boundary of the S-period to sweep through the age

FIG. 9.—Repeated labelling curves for each zone. Points show experimental data and the plotted curves are those predicted by assuming 100% proliferation (inner zone), 100% and 80% proliferation (dashed and solid lines respectively, middle zone) and 50% proliferation (outer zone).

distribution diagram). Other curves may then be generated which

(i) assume growth fractions of less than 100%.
(ii) take cell migration into account, using the results of Fig. 6.

Such curves were then compared with the experimental points. Curves which assume growth fractions of 100%, 80% and 50% in the inner, middle and outer zones respectively give a good fit to the experimental points (Fig. 9).

*Cell death*

In tumours which contain large volumes of necrotic tissue, the rates of cell death and dead cell resorption strongly influence tumour growth.

An equation which allows the rate of cell loss from a population to be estimated has been given by Steel (1967). If rate of cell loss is expressed as a fraction ($\emptyset$) of the rate of entry of cells into mitosis, then

$$\emptyset = 1 - \frac{T_{\text{pot}}}{T_{\text{true}}}$$

where

(i) $T_{\text{pot}}$ is the potential doubling time (about 26 hours for the tumour cords, Table II).
(ii) $T_{\text{true}}$ is the true doubling time (about 9·6 days for the tumour cords).

The relative rate of cell death from the cords is thus about 90%. ($\emptyset = 0{\cdot}9$).

An approximate estimate of the rate of resorption of dead cells may be obtained by comparing the rate of enlargement of the necrotic region with the rate of cell death into it. In larger tumours necrotic tissue occupies a mean of 60–70% of the tumour by volume and increases only slightly with tumour weight. The mean doubling time for necrotic enlargement is thus not substantially different from that of the tumour (i.e., 3·2 days). In tumours of similar size the cords occupy about 20% of the tumour by volume, so that within 26 hours (the turnover time for a tumour cord) the necrotic volume is increased by about one third of its original volume. Hence the measured and potential doubling times of the necrotic region are similar (i.e., about 3·2 days).

The above calculation is based on quantities which show large inter-tumour variations and is not therefore very precise. However, it appears that the growth of this tumour is characterised by a high rate of cell death, but a low resorption rate for dead tissue. This implies that an attempt to speed up the resorption process might be a better approach to controlling growth of this tumour than one which seeks to increase the rate of cell death.

### *The Influence of Oxygen*

Cell death at the periphery of a tumour cord probably occurs because the concentration of an essential metabolite falls below a critical value. Thomlinson and Gray (1955) investigated human tumours which showed a corded structure, and found a cord radius consistent with a theory of limited oxygen diffusion. Goldacre and Sylvén (1962) estimated a similar value for the oxygen diffusion length (150 $\mu$) by considering diffusion of a dye into the necrotic centre of tumours. A third and similar estimate was made by Rajewsky (1965), who demonstrated that the depth of *in vitro* labelling of tumours with tritiated thymidine depended on the oxygen tension of the medium. Caspersson and Santesson (1942), however, summarised evidence which suggests three possible causes for the development of necrosis in tumours. Cells may die from lack of glucose, from lack of oxygen, or from an injurious effect of high concentrations of lactic acid.

*Mathematical treatment of diffusion*

Adopting a notation similar to that of Thomlinson and Gray (1955), let:

$C$ = Concentration of metabolite at radius " $r$ "
$C_0$ = Concentration of metabolite next to the axial blood vessel
$M$ = Metabolite consumed per unit volume per second
$R$ = Radius at which the concentration of the metabolite falls to zero. (" the diffusion length ")
$a$ = Radius of axial blood vessel
$b$ = Tumour cord radius.

The steady state diffusion equation for cylindrical co-ordinates takes the form:

$$D\left(\frac{d^2C}{dr^2} + \frac{1}{r}\frac{dC}{dr}\right) - M = 0 \tag{1}$$

This equation may only be solved if the form of the function " $M$ " is known and it is often assumed that this is independent of metabolite concentration ($M = M_0$). For nutrient metabolites ($M$ positive), the solution must satisfy the boundary conditions.

$$\text{(i) } C = C_0 \text{ at } r = a$$

$$\text{(ii) } C = \frac{dC}{dr} = 0 \text{ at } r = R$$

The required solution is then (Forster, 1963):

$$C = C_0 + \frac{M_0}{4D}\left[(r^2 - a^2) - 2R^2 \log \frac{r}{a}\right] \tag{2}$$

and if terms " $a^2/R^2$ " are neglected, $R$ is given by

$$C_0 = \frac{M_0}{4D} R^2 \left(2 \log \frac{R}{a} - 1\right) \tag{3}$$

If now $R_0 = \sqrt{\frac{4DC_0}{M_0}}$ this becomes

$$R_0 = R\sqrt{(2 \log R/a - 1)} \tag{4}$$

The mean radius " $a$ " of tumour blood vessels has been estimated (9·5 $\mu$), so that if $R_0$ is known, the diffusion length ($R$) may be estimated graphically.

For katabolite production ($M$ negative) the second boundary condition must be replaced by

$$\frac{dC}{dr} = 0 \text{ at } r = b \ (b = \text{cord radius})$$

The solution is then

$$C = C_0 - \frac{M}{4D}\left[2b^2 \log r/a - (r^2 - a^2)\right] \tag{5}$$

This equation may be used to calculate variations in the concentration of katabolites between the centre and periphery of tumour cords.

*Computed diffusion lengths*

In the present investigation, data have been collected from various sources to estimate diffusion lengths from capillaries for oxygen and glucose (55 $\mu$ and 280 $\mu$ respectively, Tables III and IV). Variations in the concentration of lactic acid

TABLE III.—*Derivation of the Oxygen Diffusion Length*

| | Value | Reference |
|---|---|---|
| Oxygen tension in venous blood | 40 mm.Hg. | — |
| Solubility of oxygen in water at 37° C. | ·024 ml. $O_2$ at N.T.P./ml. water | Kaye, G. W. C. and Laby, T. H. (1956) |
| Water content of soft tissue (by weight) | 75% | Biology Data Book (1964) |
| $C_0$ (derived) | $9 \cdot 5 \times 10^{-4}$ ml. $O_2$ at N.T.P./ml. tissue | |
| Mean $Qo_2$ for mouse carcinomas | $15 \cdot 5$ $\mu$l.$O_2$/mg. dry wt./hr. | Standard values in nutrition and metabolism (1954) |
| $M_0$ (derived) | $1 \cdot 08 \times 10^{-3}$ ml. $O_2$ at N.T.P./ml. tissue/sec. | |
| Diffusion coeff. in aqueous soln. (D) | $1 \cdot 98 \times 10^{-5}$ cm.²/sec. | "Electrochemical Data" by Conway, B. E. (1952) |
| $R_0 = \sqrt{\frac{4DC_0}{M_0}}$ | 83 $\mu$ | |
| Diffusion length $R$ [eqn. (4)] | 55 $\mu$ | |

TABLE IV.—*Derivation of the Diffusion Length for Glucose*

| | Value | Reference |
|---|---|---|
| Mean concentration of glucose in mouse blood ($C_0$) | 174 mg./100 ml. blood | "Standard values in blood" (1952) |
| Rate of glucose consumption ($M_0$) | $3 \cdot 15$ g./hr./100 g. wet wt. tissue | Gullino *et al.* (1967)* |
| Diffusion coefficient (D) | $0 \cdot 56 \times 10^{-5}$ cm.²/sec. | "Electrochemical Data" by Conway, B. E. (1952) |
| $R_0 = \sqrt{\frac{4DC_0}{M_0}}$ | 670 $\mu$ | |
| Diffusion length $R$ [eqn. (4)] | 280 $\mu$ | |

* Estimate based on the same ratio of oxygen to glucose consumption as determined for transplanted rat tumours.

and carbon dioxide between centre and periphery of tumour cords were also estimated (21% and 16% of blood concentration respectively, Tables V and VI). In some cases the data on which these estimates are based show wide ranges and the means are not therefore very precise. In particular, the rate of oxygen consumption ($Qo_2$) has been measured for *in vitro* systems and shows wide variations among different mouse carcinomas, while histological artefacts make it difficult to estimate the capillary radius precisely. The measured mean tumour cord radius was about 90 $\mu$ and this is consistent with the calculated oxygen diffusion length to

TABLE V.—*Variations in the Concentration of Lactic Acid in Tumour Cords*

| | Value | Reference |
|---|---|---|
| Mean rate of production of lactic acid | $1 \cdot 4$ g./hr./100 g. wet wt. tissue | Gullino *et al.* (1967)* |
| Diffusion coefficient (D) | $0 \cdot 9 \times 10^{-5}$ cm.$^2$/sec. | "Electrochemical Data" by Conway, B. E. (1952) |
| Mean conc. of lactic acid in blood ($C_0$) | 13 mg./100 ml. blood | "Standard values in blood" (1952)† |
| Percent varn. in lactic acid concentration $\left(\frac{C}{C_0} \times 100\right)$ | 21% | |

* Estimate based on the same ratio of glucose consumption to lactic acid production as determined for transplanted rat tumours.

† Value for rat blood. Similar values were recorded for other species, but no estimate for mice was available.

TABLE VI.—*Variations in the Concentration of Carbon Dioxide in Tumour Cords*

| | Value | Reference |
|---|---|---|
| Rate of production of carbon dioxide, (Based on R.Q. = 1). | $1 \cdot 08 \times 10^{-3}$ ml. $CO_2$ at N.T.P./ ml. tissue/sec. | Table III |
| Diffusion coefficient (D) | $1 \cdot 71 \times 10^{-5}$ cm.$^2$/sec. | "Electrochemical Data" by Conway, B. E. (1952) |
| Water content of soft tissue (by weight) | 75% | Biology Data Book (1964) |
| Solubility of carbon dioxide in water at 37° C. | $0 \cdot 6$ ml. $CO_2$ at N.T.P./ml. water | Kaye, G. W. C. and Laby, T. H. (1956) |
| $pCO_2$ of blood ($P_0$) | 40–46 mm.Hg. | |
| Percent varn. in $CO_2$ conc. $\left(\frac{P}{P_0} \times 100\right)$ | 16% | |

within the expected error. It is lower than most other published estimates because of the cylindrical geometry of the system. Since the estimates of oxygen and glucose diffusion lengths are interdependent it is probable that the latter is also an underestimate.

Glucose may be transported through tissue by mechanisms other than diffusion. Gullino, Grantham and Courtney (1967) reported much lower concentrations of glucose in the interstitial fluid of tumours than in plasma. However, they used a micro-pore sampling technique and the chamber may have been of the order of a glucose diffusion length away from the nearest blood vessel. These authors suggested that the vascular wall may be important in regulating the supply of glucose. Such an effect could markedly decrease its diffusion length, and the high consumption which they observed would then suggest active transport between the cells themselves.

Lack of relevant information makes it difficult to investigate the diffusion of other metabolites. Amino acids are necessary for protein synthesis, but in many tissues their concentration exceeds that in blood so that active transport is involved. The tumour may depend on diffusion for a supply of lipid nutrients, but if these are in short supply, may compensate by means of an increased glucose consumption. However, on the basis of the above discussion, the structure of this mouse mammary tumour is consistent with a theory of limited oxygen diffusion.

*Effects of changes of the oxygen environment*

If the structure of this tumour is limited by oxygen diffusion, then change of the blood oxygen tension of the animal should lead to a change in both tumour growth rate and in the radius of tumour cords. The oxygen dissociation curve for haemoglobin is sigmoid shaped (Winton and Bayliss, 1962), and when animals breathe air their blood becomes about 97% saturated in the lungs. Animals breathing a mixture of 60% oxygen in nitrogen saturate their blood, while animals breathing 10% oxygen in nitrogen reduce their percentage saturation to about 70% (both mixtures at atmospheric pressure). Provided that the blood haemoglobin content remains unchanged one would expect a reduction in tumour growth rate when animals are kept in 10% oxygen, but little change if the oxygen tension is raised to 60%. This hypothesis was tested experimentally.

The experimental arrangement consisted of a 30 litre tank containing the animals, through which the gas mixture was flushed for ½ hour each day. The tank also contained dry calcium chloride to maintain humidity conditions within the limits 20–60%, and the gas mixture circulated through NaOH, to remove carbon dioxide produced by respiration. The oxygen tension was maintained by means of a flexible non-porous bag (made of 0·045 inch butyl rubber) containing pure oxygen. This was connected into the circuit by means of a long thin tube, to minimise diffusion between the chambers. As carbon dioxide was removed from the circuit, an equivalent amount of oxygen was sucked in from the bag, since this remained at atmospheric pressure. The oxygen tension was measured daily with a Hersch Cell. Animals were removed for short intervals every 3 days, in order to measure the tumours and clean the tank.

Six animals (two tumours per animal) were kept under hyperbaric conditions from the second day after transplantation until they were killed on day 15. The oxygen tension remained between 50 and 65%. Tumour growth curves for this group and for a similar number of controls were plotted (Fig. 4b) and there appear to be no differences in tumour growth rate. Animals of both groups gained body weight at similar rates.

Five animals were also kept in 10% oxygen (maintained between 9 and 11%) from the second day after transplantation until they were killed on day 17. Tumour growth curves for this group and for a similar number of controls were plotted (Fig. 4a), and comparisons between the two groups are summarised in Tables VII and VIII.

TABLE VII.—*Data for Animals Maintained Under Normal and Under Hypoxic Conditions* (10% *Oxygen*)

| | Controls | Hypoxic group |
|---|---|---|
| Mean tumour wt. at death | 2·50 g. | 0·64 g. |
| Mean body wt. at time of implantation | 26·6 g. | 25·0 g. |
| Mean body wt. at time of death | 27·6 g. | 18·2 g. |
| Mean blood haemoglobin content (at death) | 10·2 g.Hb./100 ml. blood | 14·2 g.Hb./100 ml. blood |
| Tumour cord radius (mean ± S.D.) | 85·1 ± 2·2 μ | 75·5 ± 8·8 μ |

TABLE VIII.—*A Comparison of Labelling Indices in Tumours Grown in Air and in 10% Oxygen*

| | Controls in Air % | Low Oxygen Group % |
|---|---|---|
| Inner zone | $65 \pm 6$ | $50 \pm 5$ |
| Middle zone | $48 \pm 7$ | $37 \pm 8$ |
| Outer zone | $30 \pm 5$ | $23 \pm 6$ |

Both the shape of the tumour growth curve (Fig. 4a) and the increased blood haemoglobin content (Table VII) indicate an adaptation to hypoxic conditions. The period of delay in tumour growth corresponds quite closely to the period over which the animals lose body weight (about 1 week). Thus although tumour growth is considerably slower than in the control group, this may in part be due to a more general effect on the whole animal.

For evaluation of tumour cord radius, sections were cut from tumours of the two groups and slides were randomised. Ten estimates of cord radius were made in each of the twenty tumours and means and standard deviations are shown in Table VII. The mean and standard error of the difference is $9{\cdot}6 \pm 2{\cdot}9\ \mu$.

Each animal was also injected with 50 $\mu$Ci of tritiated thymidine 1 hour before death, and those animals maintained under low oxygen were returned to the tank during this interval.

Autoradiographs were prepared from tumour sections, slides were randomised, and the labelling index was determined for each zone. The labelling index was consistently lower for the tumours of mice kept in 10% oxygen (Table VIII), indicating that the oxygen tension may play an important role in controlling the rate of cellular proliferation within the tumour cords.

### DISCUSSION

Caspersson and Santesson (1942), using an ultra-violet absorption technique, noted a whole series of metabolic changes with increasing distance from blood vessels in tumours. This led them to define two categories of cell: type A was recognised by a high cytoplasmic nucleotide content and was found close to blood vessels; type B had a low cytoplasmic nucleotide content and existed near regions of necrosis. There was a continuous range of cell types representing transitions between these two extremes. Type A cells were characterised by a high rate of protein synthesis and proliferated much more rapidly than type B cells.

Other evidence is available that suggests a high rate of cell proliferation in well-vascularised tumour regions. A high labelling index adjacent to blood vessels has been reported (Kligerman, Heidenreich and Greene, 1962; Hendrickson and Skypeck, 1963), and has also been observed by the present author in several spontaneous and transplanted tumours.

The results of the present study lend support to a hypothesis that oxygen may have a major role in controlling the rate of cellular proliferation through the growth fraction. Its influence on the median cell cycle time appears to be small, and if, as suggested above, those cells actually in contact with blood vessels have very short cell cycle times, factors other than oxygen are probably involved. There are few experimental tumour systems which have such a convenient morphological structure as the mouse mammary tumour described here. However, since the introduction of the concept of " growth fraction " (Mendelsohn, 1962), other authors have attempted to measure the proportion of non-dividing cells in tumour populations (Steel *et al.*, 1966; Frindel *et al.*, 1967). On the basis of the above hypothesis there may be a correlation between growth fraction and the relative necrotic volume of tumours, since there is evidence that both may be caused by limitations in oxygen diffusion.

Cell proliferation may be inhibited by change from respiration to anaerobic glycolysis, since the latter is probably important in relatively hypoxic areas of tumours. Such a change causes a reduction in the rate of ATP synthesis, and might inhibit cell division by decreasing the amount of available energy. However, the results of Gullino *et al.* (1967) suggest that both respiration and glycolysis may occur in all viable parts of tumours. They reported that both glucose consumption and lactate production increased in direct proportion to the oxygen used, and that lack of oxygen blocked both of them. Thus the influence of oxygen on metabolism in tumours remains a matter of speculation, and observations reported in this paper must await explanation at a subcellular level.

### SUMMARY

A mouse mammary tumour in which viable tissue was arranged cylindrically about tumour blood vessels is described, and the techniques of thymidine autoradiography have been used to investigate parameters of cellular proliferation within these tumour cords. Both labelling and mitotic index were found to decrease with increasing distance from the axial blood vessel. Labelled mitoses and repeated labelling results suggested a decrease in the growth fraction. The median cell cycle time, however, showed little variation in the tumour cords.

The diffusion of metabolites was discussed in the light of other published data and the cord structure was found to be consistent with a theory of limited oxygen diffusion. This hypothesis was tested by investigating tumour growth in animals maintained under conditions of hyperbaric oxygen and of hypoxia. Hypoxic conditions caused a reduction in both the mean tumour cord radius and in the labelling index.

I should like to thank both Professor L. F. Lamerton and Dr. G. G. Steel for their constant encouragement and useful discussion. Miss J. Lambert provided valuable technical assistance throughout the project, and I should like to record my thanks to her, and the many other members of the Department who advised or assisted at various times.

### REFERENCES

Barrett, J. C.—(1966) *J. natn. Cancer Inst.*, **37**, 443.
'Biology Data Book'—(1956) Edited by P. L. Althan and D. S. Ditther. Washington (Federation of American Societies for Experimental Biology).
Caspersson, T. and Santesson, L.—(1942) *Acta Radiol.*, Suppl. 46.
Chalkley, H. W.—(1943) *J. natn. Cancer Inst.*, **4**, 47.
Conway, B. E.—(1952) 'Electrochemical data'. Amsterdam (Elsevier).
Forster, R. E.—(1963) 'Oxygen in the animal organism'. Edited by F. Dickens and E. Neil. *Int. Symp. IUB/IUBS*, Vol. 31, p. 393.
Frindel, E., Malaise, E. P., Alpen, E. and Tubiana, M.—(1967) *Cancer Res.*, **27**, 1122.

GOLDACRE, R. J. AND SYLVÉN, B.—(1962) *Br. J. Cancer*, **16**, 306.
GULLINO, P. M., GRANTHAM, F. H. AND COURTNEY, A. H.—(1967) *Cancer Res.*, **27**, 1020, 1031, 1041.
HENDRICKSON, F. R. AND SKYPECK, H.—(1963) *Radiology*, **80**, 244.
KAY, G. W. C. AND LABY, T. H.—(1956) 'Tables of physical and chemical constants'. London (Longman's Green & Co.)
KLIGERMAN, M. M., HEIDENREICH, W. F. AND GREENE, S.—(1962) *Nature, Lond.*, **196**, 282.
MENDELSOHN, M. L.—(1962) *J. natn. Cancer Inst.*, **28**, 1015.
RAJEWSKY, M. F.—(1965) *Eur. J. Cancer*, **1**, 281.
'STANDARD VALUES IN BLOOD'—(1952) Edited by E. C. Albriton. Philadelphia and London (W. B. Saunders).
'STANDARD VALUES IN NUTRITION AND METABOLISM'—(1954) Edited by E. C. Albriton. Philadelphia and London (W. B. Saunders).
STEEL, G. G.—(1967) *Eur. J. Cancer*, **3**, 381.
STEEL, G. G., ADAMS, K. AND BARRETT, J. C.—(1966) *Br. J. Cancer*, **20**, 784.
THOMLINSON, R. H. AND GRAY, L. H.—(1955) *Br. J. Cancer*, **9**, 539.
WINTON, F. R. AND BAYLISS, L. E. (1962) 'Human physiology', 5th edition. London (Churchill) p. 77.